# Association of Serum Calcium with the Risk of Chronic Obstructive Pulmonary Disease: A Prospective Study from UK Biobank

**DOI:** 10.3390/nu15153439

**Published:** 2023-08-03

**Authors:** Xinglin Wan, Lulu Chen, Zheng Zhu, Pengfei Luo, Dong Hang, Jian Su, Ran Tao, Jinyi Zhou, Xikang Fan

**Affiliations:** 1 Department of Epidemiology, School of Public Health, Nanjing Medical University, Nanjing 211166, China; wanxl19981010@163.com (X.W.); hangdong@njmu.edu.cn (D.H.); trltjy@163.com (R.T.); 2 Department of Non-Communicable Chronic Disease Control, Jiangsu Provincial Center for Disease Control and Prevention, Nanjing 210009, China; chenlulu136@163.com (L.C.); zhengzhu@jscdc.cn (Z.Z.); lpfei215@live.cn (P.L.); sujiangx@163.com (J.S.)

**Keywords:** chronic obstructive pulmonary disease, serum calcium, cohort study, incidence, mortality

## Abstract

Background: Although intracellular calcium had been demonstrated to involve in the pathogenesis of chronic obstructive pulmonary disease (COPD), the association between serum calcium and COPD risk remains unclear. Methods: We included 386,844 participants with serum calcium measurements and without airway obstruction at the baseline from UK Biobank. The restricted cubic splines were used to assess the dose–response relationship. Multivariable cox regression models were used to estimate hazard ratios (HRs) and 95% confidence intervals (CIs) for the associations of albumin-corrected calcium concentrations with the risk of COPD incidence and mortality. Results: During a median of 12.3 years of follow-up, 10,582 incident COPD cases were documented. A linear positive association was observed between serum calcium concentrations and the risk of COPD incidence. Compared to participants with normal serum calcium (2.19–2.56 mmol/L), a 14% higher risk of COPD was observed in hypercalcemic participants (≥2.56 mmol/L, HR = 1.14; 95% CI: 1.02–1.27). No significant effect modifications were observed in stratified variables. In survival analysis, 215 COPD-specific deaths were documented after a median survival time of 3.8 years. Compared to participants with normal serum calcium, hypercalcemic participants had a 109% (HR = 2.09, 95% CI: 1.15–3.81) increased risk for COPD-specific mortality. Conclusion: Our study indicated that hypercalcemia was associated with an elevated risk of COPD incidence and mortality in the European population, and suggested that serum calcium may have a potential impact on the progression of COPD.

## 1. Introduction

Calcium, the single most abundant mineral in the human body, exists primarily in the bone as hydroxyapatite and only a small fraction is dissolved in body fluids as ions [1]. Traditionally, calcium has been well-known for its effects on bone health [2], but calcium ions in body fluids also play an essential role in many physiological functions. Numerous studies have shown that calcium ions are involved in physiological processes such as cell signaling [3], muscle contraction [4], and neurotransmission [5]. The dynamic balance of calcium ion concentrations between intracellular and extracellular fluids is regulated by calcium channels in the cell membrane [6]. When this homeostasis is deregulated, it can lead to the development of a variety of diseases [7].

Chronic obstructive pulmonary disease (COPD) is a leading cause of chronic morbidity and mortality worldwide. Based on the Global Burden of Disease Study, COPD incidence and mortality rates in 2019 were 200.49/100,000 and 42.52/100,000 persons, respectively [8]. COPD is a heterogeneous lung condition characterized by persistent and often progressive airflow obstruction, resulting from the interaction of various risk factors, including genetics, age, smoking, and air pollution [9]. Recently, a plethora of studies explored biomarkers to aid in the early prevention and treatment of COPD [10]. Existing mechanistic studies have identified an important role for intracellular calcium ion concentrations in the pathogenesis of COPD [11]. However, few epidemiology studies explore the association between serum calcium concentrations and the development of COPD.

Several population-based cohort studies have led to observations of associations between serum calcium concentrations and the risk of all-cause, CVD-specific, and cancer-specific mortality [12,13]. Mechanistic studies have also found that the dysregulation of intracellular calcium homeostasis in COPD patients often induces pulmonary hypertension, which leads to death [14]. Nevertheless, the impact of prediagnostic serum calcium levels on COPD prognosis remains unclear.

Therefore, leveraging data from the UK Biobank dataset, this study aimed to assess the associations of serum calcium concentrations with the risk of COPD incidence and mortality in the European population.

## 2. Materials and Methods

### 2.1. Study Population

UK Biobank is a large prospective study that aims to improve the prevention, diagnosis, and treatment of a wide range of diseases. The study recruited over 500,000 participants aged between 40 and 69 years old from across the United Kingdom between 2006 and 2010. Participants provided extensive sociodemographic characteristics, health information, and a wide range of biological samples. All participants signed consent forms and were registered with the UK National Health Service (NHS). The start of the follow-up for each participant was the date of completion of their baseline assessment. The present study was conducted under UK Biobank application number 84525.

We excluded 49,733 participants with airway obstruction, which included 3516 individuals with COPD at recruitment, 8349 individuals with self-reported emphysema/chronic bronchitis, 41,779 individuals with a forced expiratory volume at a 1 s/forced vital capacity (FEV_1_/FVC) lower than the lower limit of the normal (LLN) value calculated according to the Global Lung Function Initiative (GLI) 2012 criteria [15]. Furthermore, 65,836 participants with unavailable measurements for serum calcium or albumin were excluded. In total, 386,844 participants were included in the final analysis (Supplementary Figure S1).

### 2.2. Assessment of Biomarkers

Serum calcium concentrations were measured using a colorimetric assay on the Beckman Coulter AU5800 platform, with a detection range of 1–5 mmol/L. The average coefficients of variation were 1.29%, 1.39%, and 1.61% for low, medium, and high internal quality control (IQC) levels of serum calcium. The assay was registered with an external quality assurance scheme (EQA), which showed that 100% of sample distributions were good or acceptable. Serum albumin concentrations were also measured on the same platform using a bromocresol green method. Since serum calcium concentrations vary with albumin levels, we calculated albumin-corrected calcium using a formula based on the UK Biobank population: corrected calcium (mmol/L) = total calcium (mmol/L) + 0.0177 [45.2 (g/L)—albumin (g/L)] [16]. Further details on the procedures for analyzing creatinine and 25-hydroxyvitamin D [25(OH)D] can be found in the UK Biobank showcase [https://biobank.ndph.ox.ac.uk/showcase/ (accessed on 2 July 2023)].

### 2.3. Ascertainment of Outcomes

We used ICD-10 (the 10th revision of the International Classification of Diseases) codes to define COPD as J40-J44. Incident COPD cases during follow-up were identified through linkage to the NHS register. The hospital admission data for the current analysis were censored on 30 September 2021 for England, 31 July 2021 for Scotland, and 28 February 2018 for Wales. The cause and date of death after COPD diagnosis were obtained from death certificates until 31 October 2021 for Scotland and 30 September 2021 for England and Wales.

### 2.4. Ascertainment of Covariates

Baseline variables including demographic information, lifestyle, and medical history were derived from participants who completed questionnaires. Chronological age was calculated from the date of birth to the baseline assessment. We defined fasting status according to the time between the last meal and the blood draw (defining ≥8 h as fasting, and <8 h as non-fasting). Each participant was assigned a Townsend deprivation index corresponding to the postcode of residence. We calculated the body mass index (BMI, kg/m^2^) as weight (kg) divided by height (m) squared. Physical activity was based on self-reported moderate activity and measured as a metabolic equivalent task (MET, hours/week). We calculated the estimated glomerular filtration rate (eGFR) according to the Chronic Kidney Disease Epidemiology Collaboration creatinine equation [16]. Prevalent asthma was defined using self-report and hospital admission data (ICD-10 codes J45-J46). The UK Biobank online protocol was used to record details on baseline variable measurements [17].

### 2.5. Statistical Analysis

Participants were divided into groups of hypocalcemia (<2.19 mmol/L), normal serum calcium (2.19–2.56 mmol/L), or hypercalcemia (≥2.56 mmol/L) according to the reference interval for albumin-corrected calcium concentrations in the UK Biobank population [18]. Baseline characteristics were described among participants grouped by reference interval and deciles of calcium concentrations. Participants’ person-time was considered to be from the date of the assessment until the date of the initial diagnosis of COPD, being lost to follow-up, death, or the end of the study, whichever occurred first.

We used multivariable restricted cubic splines with four knots to explore dose–response relationship between serum calcium concentrations and the risk of COPD incidence. To further assess the association between serum calcium and the risk of COPD, we used multivariable cox proportional hazard models to estimate the hazard ratios (HRs) and 95% confidence intervals (CIs). The proportional hazards assumption was tested using Schoenfeld residuals and no violation of the assumption was detected.

We classified serum calcium into deciles and three groups according to the reference interval and estimated the HRs using the fifth decile or normal group as a reference. To reflect a gradual increase in the serum calcium concentration and follow a normal distribution, we also divided the serum calcium concentrations by the standard deviation (SD) to calculate the HRs per 1 SD increase and *p* for the trend. Two analytic models were considered: the basic model adjusted for age, sex (female, male), race (white, non-white), fasting status (<8 h, ≥8 h), and assessment centers, and the fully adjusted model which further included possession of a college or university degree (yes or no), the Townsend deprivation index, BMI, smoking status (never, previous, or current), passive smoking (never, <20 and ≥20 h a week), alcohol drinking (never or special occasions only, once a month to twice a week, and three times a week to daily), physical activity, prevalent asthma (yes or no), family history of respiratory diseases (yes or no), eGFR, 25(OH)D, PM_2.5_, and occupations at risk of COPD (yes or no). For covariates with missing data, we used the missing indicator method for categorical variables and used median imputation by gender for continuous variables.

To test the robustness of the primary analysis, we conducted sensitivity analyses by excluding incident COPD cases within the first two years of follow-up, excluding the participants with overall poor self-rated health in the baseline questionnaires, and additionally adjusting female-specific factors. Moreover, stratified analyses were conducted by age (≤60 to >60 years), sex (male or female), BMI category (<25, 25–30, or ≥30 kg/m^2^), smoking status (never, former, and current smokers), passive smoking (never or ever), 25(OH)D level (<50, ≥50 nmol/L), eGFR [<90, ≥90 mL/(min × 1.73 m^2^)] in the fully adjusted model. We performed a likelihood ratio test to examine possible effect modifications between serum calcium concentrations and stratified variables by comparing the models with and without interaction terms.

In the survival analyses, we computed the overall and COPD-specific survival time from the initial COPD diagnosis until death or the end of the follow-up. Restricted cubic splines with four knots were constructed to evaluate dose–response relationships between serum calcium concentrations and the risk of COPD mortality. Moreover, we performed multivariate cox regression analysis to estimate the HRs for the associations of serum calcium with all-cause and COPD-specific mortality. Our survival analyses were adjusted for the same covariates as carried out in previous analyses.

We performed the all above analyses using SAS 9.4 (SAS Institute, Inc., Cary, NC, USA). Statistical significance was defined by a two-sided *p* of < 0.05.

## 3. Results

During a median follow-up of 12.3 (interquartile range, 11.4–13.2) years, 10,582 (5664 in males, and 5918 in females) incident COPD cases were recorded. Among these COPD cases, we documented 2662 deaths (215 from COPD) within a median survival time of 3.8 (interquartile range: 1.6–6.6) years. Table 1 presents the baseline characteristics of participants grouped by the normal range and Supplementary Table S1 presents the deciles of serum calcium concentrations. Participants with higher calcium concentrations were more likely to be elderly, have a high education degree, intense physical activity, a family history of respiratory diseases, high 25(OH)D concentrations, and a low eGFR.

**Figure 1:**
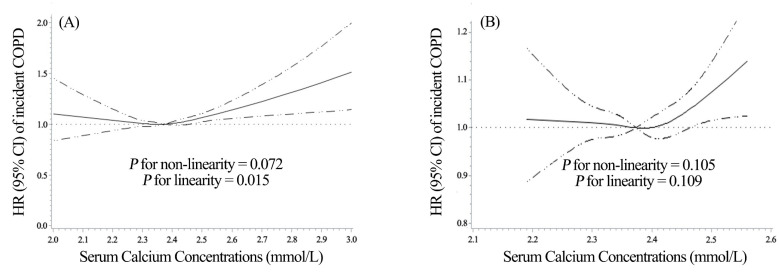
Dose–response relationships between albumin-corrected calcium concentrations and the risk of COPD incidence in all participants (**A**) and participants with normal calcium concentrations (**B**). The associations were examined via multivariate cox regression models based on restricted cubic splines. Median serum calcium concentrations serve as a reference. The solid line represents estimates of hazard ratios and the dashed lines represent 95% confidence intervals.

Figure 1A revealed a linear positive relationship between serum calcium concentrations and the risk of COPD incidence in all participants (*p* for non-linearity = 0.072 and *p* for linearity = 0.015). However, there was no statistically significant dose–response relationship among participants with normal calcium concentrations (*p* for non-linearity = 0.105 and *p* for linearity = 0.109) (Figure 1B). Table 2 shows the associations between serum calcium and COPD incidence among participants classified by deciles of calcium concentrations and the normal range. In the fully adjusted model, compared to participants in the fifth decile, those in the highest decile had a 10% increased risk of COPD incidence (HR = 1.10; 95% CI = 1.01–1.20). Participants in the hypercalcemic group had a 14% increased risk of COPD (HR = 1.14; 95% CI = 1.02–1.27) incidence compared to those in the normal serum calcium group. Moreover, the result of the trend test showed that the risk of COPD increased by 2% per 1 SD increase in serum calcium concentrations (HR = 1.02, 95% CI: 1.00–1.04).

Stratified analysis showed that the associations between serum calcium and the risk of COPD incidence remained consistent across the subgroup (Supplementary Figure S2). Moreover, sensitivity analyses suggested that the aforementioned association was unchanged after excluding incident COPD cases within the first two years of follow-up (*n* = 789), excluding participants with poor self-rated health at the baseline (*n* = 15,248), and additionally adjusting for female-specific factors (Supplementary Table S2).

Supplementary Figure S3 showed a linear positive relationship between serum calcium concentrations and the risk of all-cause mortality among incident COPD cases (*p* for non-linearity = 0.116 and *p* for linearity < 0.0001), but no significant relationship was found with COPD-specific mortality (*p* for non-linearity = 0.267 and *p* for linearity = 0.261). Compared to the normal group, participants in the hypercalcemic group had an increased risk of all-cause (HR = 1.24; 95% CI = 1.01–1.53) and COPD-specific mortality (HR = 2.09; 95% CI = 1.15–3.81). The trend test revealed a 9% increase in the risk of all-cause mortality in COPD cases per one SD increase in serum calcium (HR = 1.09; 95% CI = 1.05–1.13) (Table 3).

## 4. Discussion

In this prospective study, 10,582 cases of incident COPD occurred in participants and subsequently 2662 cases resulted in death. We observed for the first time a positive linear dose–response relationship of albumin-corrected calcium concentrations with the risk of COPD incidence in all participants and all-cause mortality among incident COPD cases. In addition, we found that hypercalcemic participants have an elevated risk of COPD incidence and mortality. Our study implies the potential impact of serum calcium on the progression of COPD.

To the best of our knowledge, no previous studies have examined the association between serum calcium and the risk of COPD. COPD is a chronic inflammatory disease that causes chronic obstructive bronchiolitis and emphysema [19]. The major pathophysiology of COPD development involves airway smooth muscle cell (ASMC) remodeling and dysfunction. Altered contractile function, airway hyper-responsiveness (AHR), and an excessive proliferation of the ASMC are the key features of the development of COPD [20]. However, elevated intracellular calcium has been demonstrated to contribute to the hyperproliferation and hypertrophy of ASMC in COPD [21]. Smoking is a recognized risk factor for COPD, and nicotine in tobacco smoke binds with nicotinic acetylcholine receptors (nAChRs) on bronchial epithelial cells (BEC) and leads to an increased intracellular calcium influx [22]. Sassano et al. found that HEK293T cells exposed to tobacco smoke or metabolites increase intracellular calcium levels [23]. Nicotine has been proven to increase the calcium influx in the BEC by activating protein kinase C, p38 MAPK pathways, or the Erk1/2 pathway in fibroblasts [24]. The elevation of intracellular calcium concentrations is one of the key mechanisms regulating ASMC contractility through the G protein-coupled receptor (GPCR) pathway, transient receptor potential (TRP) channels, and store-operated Ca^2+^ channels (SOCC) [25,26,27]. Among these, there was an enhanced expression of TRP3 channels (a subtype of TRP channels) and α5-nAChR induced ASMC proliferation and a calcium influx. An imbalance in calcium transport could lead to dysregulated ASMC function and further COPD development [11]. Moreover, several experimental studies suggest that reducing the ionized calcium influx to modulate ASMC contractility may be a potential therapeutic strategy for COPD [28,29]. 

Our study for the first time indicated that hypercalcemia might contribute to a higher risk of COPD incidence. However, there is still a lack of detailed mechanism studies on how serum calcium concentrations affect the development of COPD. Calcium homeostasis has a vital role in the maintenance of respiratory health [30,31]. High serum calcium concentrations might cause elevated intracellular calcium levels and thus lead to dysregulated ASMC function, and further COPD development [11]. Therefore, the role of serum calcium in the calcium regulation of COPD pathogenesis needs to be further investigated in future studies.

Existing epidemiologic evidence suggests that serum calcium concentrations are associated with all-cause mortality in the population [32,33]. A recent prospective study based on UK Biobank and the National Health and Nutrition Examination Survey (NHANES) found a U-shaped relationship between serum calcium concentrations and all-cause mortality [12]. However, another recent study from the NHANES population reported an L-shaped relationship with all-cause mortality [13]. The reason for this discrepancy in conclusion could be that the latter did not correct for albumin. Since nearly half of the serum calcium is bound to albumin [34], the use of albumin-corrected calcium in the assessment of serum calcium was highly recommended [35]. Notably, the association between serum calcium and COPD mortality has not yet been reported. Mechanistic studies showed that elevated intracellular calcium levels induced the hyperproliferation of pulmonary arterial smooth muscle cells (PASMCs), which leads to COPD-associated pulmonary hypertension and even death [36]. Our study revealed that higher albumin-corrected calcium concentrations were associated with an increased risk of all-cause and COPD-specific mortality in COPD cases. This finding might spur further research on the underlying benefits of targeting serum calcium for the treatment of COPD.

Our study has some potential strengths. First, the prospective design, large sample size, and strict exclusion of participants provided ample power to evaluate the association between serum calcium concentrations and COPD risk. Second, to minimize underlying measurement errors, standardized and validated methods were utilized to measure all serum biomarkers with strict quality control procedures. However, several limitations should be acknowledged. First, a single measurement at the baseline may not reflect long-term serum calcium levels in the follow-up period. Second, the serum calcium measurements in the UK Biobank database represent total serum calcium concentrations, but ionized calcium is the active component in the body. However, we corrected total serum calcium concentrations based on albumin to approximately match the ionized calcium level. Third, potential reverse causation could not be avoided. However, we excluded participants with airway obstruction at the baseline from the analysis to reduce the impact on the risk of COPD in the follow-up period. Finally, COPD exacerbation is a common prognosis of COPD, but our results were limited by the number of outcome cases. Follow-up outcomes were obtained through the UK National Health Service, but COPD exacerbation has a rapid onset and thus could not be registered in time.

## 5. Conclusions

Our study indicated that hypercalcemia was associated with an elevated risk of COPD incidence and mortality, and suggested that serum calcium may have a potential impact on the progression of COPD. The underlying causality and mechanisms between hypercalcemia and COPD risk may spur future clinical or trial studies.

## Figures and Tables

**Table 1:** Baseline characteristics of participants according to the normal range of albumin-corrected calcium concentrations.

Characteristics	Albumin-Corrected Calcium Concentrations (mmol/L)			
	Total	Hypocalcemia (<2.19)	Normal (2.19–2.56)	Hypercalcemia (≥2.56)
N	386,844	2272	375,897	8675
Age, mean (SD), y	56.48 (8.1)	54.82 (8.5)	56.43 (8.1)	59.30 (7.0)
Follow-up time, mean (SD), y	11.75 (2.2)	11.76 (2.4)	11.76 (2.2)	11.53 (2.3)
Female, No. (%)	209,724(54.2)	994 (43.8)	202,191 (53.8)	6539 (75.4)
White race, No. (%)	365,358 (94.5)	2097 (92.5)	355,128 (94.6)	8133 (93.8)
College or university degree, No. (%)	127,174 (32.9)	893 (39.4)	123,971 (33.0)	2310 (26.7)
Fasting before blood draw, No. (%)	15,471 (4.0)	149 (6.6)	15,051 (4.0)	271 (3.1)
Townsend index, mean (SD)	−1.39 (3.0)	−1.00 (3.2)	−1.39 (3.0)	−1.34 (3.0)
Smoking status, No. (%) ^a^				
Never	216,063 (55.9)	1323 (58.3)	209,932 (55.9)	4808 (55.5)
Previous	133,061 (34.4)	734 (32.4)	129,290 (34.4)	3037 (35.0)
Current, pack-years <10	4811 (1.2)	32 (1.4)	4704 (1.3)	75 (0.9)
Current, pack-years ≥10 and <20	6799 (1.8)	30 (1.3)	6623 (1.8)	146 (1.7)
Current, pack-years ≥20 and <30	6264 (1.6)	39 (1.7)	6070 (1.6)	155 (1.8)
Current, pack-years ≥30	9853 (2.6)	44 (1.9)	9560 (2.6)	249 (2.9)
Passive smoking, No. (%) ^a^				
Never	257,510 (71.4)	1510 (71.0)	250,372 (71.4)	5628 (69.7)
<20 h a week	69,187 (19.2)	430 (20.2)	67,207 (19.17)	1550 (19.2)
≥20 h a week	4229 (1.2)	28 (1.3)	4115 (1.2)	86 (1.1)
PM_2.5_, mean (SD), μg/m^3^	9.97 (1.1)	10.02 (1.1)	9.97 (1.1)	9.97 (1.0)
Alcohol intake frequency, No. (%) ^a^				
Never or special occasions only	73,738 (19.1)	448 (19.8)	70,983 (18.9)	2307 (26.6)
Once a month to twice a week	143,781 (37.2)	828 (36.5)	139,715 (37.2)	3238 (37.4)
Three times a week to daily	168,518 (43.6)	989 (43.6)	164,422 (43.8)	3107 (35.9)
Body mass index, mean (SD), kg/m^2^	27.50 (4.8)	27.31 (5.0)	27.48 (4.8)	27.99 (5.1)
eGFR, mL/min/1.73 m^2^	90.89 (13.4)	94.12 (16.1)	90.97 (13.30)	86.55 (14.9)
25(OH)D, nmol/L	48.72 (21.0)	43.41 (20.7)	48.74 (21.0)	49.54 (21.0)
Physical activity, mean (SD), MET-hour/week	15.54 (20.4)	13.97 (18.8)	15.55 (20.4)	15.77 (20.5)
Family history of respiratory disease, No. (%)	57,328 (14.8)	309 (13.6)	55,532 (14.8)	1487 (17.1)
Prevalent Asthma, No. (%)	39,142 (10.1)	221 (9.7)	37,972 (10.1)	949 (10.9)
Occupations associated with COPD, No. (%)	7259 (1.9)	48 (2.1)	7029 (1.9)	182 (2.1)

^a^: The percentage of the categorical variables does not amount to 100% because some participants chose to “not answer”. Abbreviations: MET, metabolic equivalent; PM_2.5_, fine particulate matter; eGFR, estimated glomerular filtration rate; 25(OH)D, 25-hydroxyvitamin D; COPD, chronic obstructive pulmonary disease.

**Table 2:** Associations of albumin-corrected calcium concentrations with the risk of COPD.

Groups	No. of COPD /Person-Years	Basic Model ^a^	Fully Adjusted Model ^b^
Deciles of albumin-corrected calcium concentrations (mmol/L)			
Decile 1 (1.12–2.28)	916/458,796	0.96 (0.88–1.05)	1.02 (0.93–1.12)
Decile 2 (2.28–2.31)	943/459,001	0.96 (0.88–1.05)	1.00 (0.92–1.10)
Decile 3 (2.31–2.34)	977/456,543	1.00 (0.91–1.09)	1.04 (0.95–1.14)
Decile 4 (2.34–2.36)	955/456,728	0.96 (0.88–1.05)	0.98 (0.90–1.07)
Decile 5 (2.36–2.38)	1000/456,149	ref	ref
Decile 6 (2.38–2.39)	1071/454,612	1.06 (0.97–1.15)	1.05 (0.96–1.14)
Decile 7 (2.39–2.41)	1063/453,479	1.04 (0.96–1.14)	0.99 (0.91–1.08)
Decile 8 (2.41–2.44)	1085/452,444	1.06 (0.98–1.16)	1.00 (0.91–1.09)
Decile 9 (2.44–2.48)	1237/450,688	1.20 (1.10–1.30)	1.07 (0.98–1.16)
Decile 10 (2.48–3.56)	1335/448,147	1.25 (1.15–1.36)	1.10 (1.01–1.20)
Reference interval for UK Biobank population (mmol/L)			
Hypocalcemia (<2.19)	59/26,723	1.02 (0.79–1.32)	1.07 (0.83–1.39)
Normal (2.19–2.56)	10,188/4,419,880	ref	ref
Hypercalcemia (≥2.56)	335/99,983	1.30 (1.16–1.45)	1.14 (1.02–1.27)
*p* for trend		<0.0001	0.020
HR per 1-SD increment ^c^		1.09 (1.07–1.11)	1.02 (1.00–1.04)

^a^ Basic model: adjusted for age, sex, ethnicity, assessment centers, and fasting status. ^b^ Fully adjusted model: adjusted for basic model plus college or university degree, body mass index, smoking status and pack-years, alcohol drinking, summed metabolic equivalent of task-minutes per week for activity, family history of respiratory diseases, prevalent asthma, estimated glomerular filtration rate (eGFR), 25(OH)D, passive smoking, PM_2.5_, and occupations associated with COPD. ^c^ The SD was 0.082 mmol/L for albumin-adjusted serum calcium. Abbreviations: HR, hazard ratio; CI, confidence interval; ref, reference.

**Table 3:** Associations of albumin-corrected calcium concentrations with the risk of all-cause and COPD-specific mortality.

Model	Reference Interval for UK Biobank Population (mmol/L)				
	Hypocalcemia (<2.19)	Normal (2.19–2.56)	Hypercalcemia (>2.56)	*p* for Trend	HR per 1-SD Increment ^c^
All-cause					
death cases/total cases	21/59	2544/10,188	97/335		
Basic model ^a^	1.19 (0.77–1.83)	Ref	1.29 (1.05–1.58)	<0.0001	1.09 (1.05–1.13)
Fully-adjusted model ^b^	1.23 (0.80–1.89)	Ref	1.24 (1.01–1.53)	<0.0001	1.09 (1.05–1.13)
COPD-specific					
death cases/total cases	3/59	200/10,188	12/335		
Basic model ^a^	1.89 (0.60–5.97)	Ref	2.17 (1.20–3.90)	0.343	1.07 (0.93–1.22)
Fully-adjusted model ^b^	1.80 (0.56–5.78)	Ref	2.09 (1.15–3.81)	0.254	1.08 (0.94–1.24)

^a^ Basic model: adjusted for age, sex, ethnicity, assessment centers, and fasting status. ^b^ Fully-adjusted model: adjusted for basic model plus college or university degree, body mass index, smoking status and pack-years, alcohol drinking, summed metabolic equivalent of task-minutes per week for activity, family history of respiratory diseases, prevalent asthma, estimated glomerular filtration rate (eGFR), 25(OH)D, passive smoking, PM_2.5_, and occupations associated with COPD. ^c^ The SD was 0.086 mmol/L for albumin-adjusted serum calcium. Abbreviations: HR, hazard ratio; CI, confidence interval; ref, reference.

## Data Availability

The UK Biobank data are available from the UK Biobank upon request [www.ukbiobank.ac.uk/ (accessed on 2 July 2023)].

## Supplementary Materials

The following supporting information can be downloaded at: https://www.mdpi.com/article/10.3390/nu15153439/s1, Figure S1: Flow chart of study participants; Figure S2: Forest plots of stratified analysis of the as-sociation between albumin-corrected calcium concentrations and risk of COPD incidence; Figure S3: Dose-response relationships between albumin-corrected calcium concentrations and the risk of all-cause (A) and COPD-specific (B) mortality in COPD cases; Table S1: Baseline characteristics of participants according to decile of albumin-corrected calcium concentrations; Table S2: Sensitivity analyses for the associations between albumin-corrected calcium and the risk of COPD incidence.
