# Supplementary material for: Association of Serum Calcium with the Risk of Chronic Obstructive Pulmonary Disease: A Prospective Study from UK Biobank
Source: Nutrients. 2023 Aug 3;15(15):3439. doi: 10.3390/nu15153439 (PMC10421293; doi:10.3390/nu15153439)
Supplement: Supplementary file 1 [file nutrients-15-03439-s001.zip › nutrients-2516471-supplementary.pdf]

Supplementary Table S1. Baseline characteristics of participants according to decile of albumin-corrected calcium concentrations

Characteristics	Albumin-corrected calcium concentrations (mmol/L)			
	Total	Decile 1 (<2.28)	Decile 5 (2.35-2.37)	Decile 10 (≥2.48)
**N**	386 844	38 684	38 686	38 685
**Age, mean (SD), y**	56.48 (8.1)	54.81 (8.5)	56.23 (8.1)	58.55 (7.4)
**Follow-up time, mean (SD), y**	11.75 (2.2)	11.86 (2.1)	11.79 (2.1)	11.58 (2.2)
**Female, No. (%)**	209 724(54.2)	17 048 (44.1)	20 216 (52.3)	26 868 (69.5
**White race**	365 358 (94.5)	36 327 (94.0)	36 646 (94.8)	36 278 (93.9)
**College or university degree, No. (%)**	127 174 (32.9)	14 899 (38.6)	12 971 (33.6)	10 595 (27.4)
**Fasting before blood draw, No. (%)**	15 471 (4.0)	2 047 (5.30)	1 512 (3.9)	1 315 (3.4)
**Townsend index, mean (SD), y**	-1.39 (3.0)	-1.29 (3.1)	-1.42 (3.0)	-1.34 (3.0)
**Smoking status, No. (%)[a]**				
**Never**	216 063 (55.9)	22 127 (57.3)	21 550 (55.8)	21 351 (55.3)
**Previous**	133 061 (34.4)	12 969 (33.6)	13 423 (34.7)	13 471 (34.9)
**Current, pack-years <10**	4811 (1.2)	507 (1.3)	517 (1.3)	396 (1.0)
**Current, pack-years ≥10 and <20**	6799 (1.8)	644 (1.7)	663 (1.7)	725 (1.9)
**Current, pack-years ≥20 and <30**	6264 (1.6)	550 (1.4)	635 (1.6)	680 (1.8)
**Current, pack-years ≥30**	9853 (2.6)	776 (2.0)	939 (2.4)	1144 (3.0)
**Passive smoking, No. (%)[a]**				
**Never**	257 510 (71.4)	26 016 (71.7)	25 808 (71.4)	25 244 (70.4)
**<20 hours a week**	69 187 (19.2)	7 135 (19.7)	7 004 (19.4)	6 758 (18.8)
**≥20 hours a week**	4 229 (1.2)	429 (1.2)	428 (1.2)	392 (1.1)
**$PM_{2.5}$, mean (SD), μg/m^3**	9.97 (1.1)	10.01 (1.7)	9.93 (1.0)	9.88 (1.0)
**Alcohol intake frequency, No. (%)[a]**				
**Never or special occasions only**	73 738 (19.1)	6 741 (17.5)	7 111 (18.4)	9 199 (23.8)
**Once a month to twice a week**	143 781 (37.2)	14 240 (36.9)	14 293 (37.0)	14 479 (37.5)
**Three times a week to daily**	168 518 (43.6)	17 595 (45.5)	17 218 (44.5)	14 923 (38.6)
**Body mass index, mean (SD), kg/m^2**	27.50 (4.8)	27.20 (4.7)	27.45 (4.7)	27.95 (5.0)
**eGFR, mL/min/1.73 m^2**	90.89 (13.4)	93.76 (13.5)	91.26 (13.00)	87.51 (14.1)
**25(OH)D, nmol/L**	48.72 (21.0)	45.43 (20.6)	48.55 (20.9)	50.83 (21.2)
**Physical activity, mean (SD), MET-hour/week**	15.55 (20.4)	14.95 (20.1)	15.65 (20.6)	15.81 (20.5)
**Family history of respiratory disease, No. (%)**	57 328 (14.8)	5 302 (13.7)	5 772 (14.9)	6 341 (16.4)
**Prevalent Asthma, No. (%)**	39 142 (10.1)	3 925 (10.2)	3 911 (10.1)	4 032 (10.4)
**Occupations associated with COPD, No. (%)**	7 259 (1.9)	723 (1.9)	709 (1.8)	783 (2.0)

[a]: The percentage of the categorical variables does not sum to 100% because some participants chose to "not answer"

Abbreviations: MET, metabolic equivalent; $PM_{2.5}$, fine particulate matter; eGFR, estimated glomerular filtration rate; 25(OH)D, 25-hydroxyvitamin D; COPD, chronic obstructive pulmonary disease;

Supplementary Table S2. Sensitivity analyses for the associations between albumin-corrected calcium and the risk of COPD incidence

Model	Reference interval for UK Biobank population (mmol/L)				
	**Hypocalcemia (<2.19)**	**Normal (2.19-2.56)**	**Hypercalcemia (>2.56)**	***P* for trend**	**HR per 1-SD increment[b]**
**The primary findings for the total cohort**					
No. of COPD/person-years	59/26 723	10 188/4 419 880	335/99 983		
Fully-adjusted model[a]	1.07 (0.83-1.39)	ref	1.14 (1.02-1.27)	0.020	1.02 (1.00-1.04)
**Excluding incident COPD cases within the first two years of follow-up (n=789)**					
No. of COPD/person-years	51/26 714	9 427/4 419 038	315/99 964		
Fully-adjusted model[a]	1.01 (0.77-1.34)	ref	1.16 (1.04-1.30)	0.008	1.03 (1.01-1.05)
**Excluding participants with poor self-rated health (n=15 248)**					
No. of COPD/person-years	51/25 476	8 656/4 261 861	278/95 302		
Fully-adjusted model[a]	1.14 (0.87-1.51)	ref	1.12 (0.99-1.26)	0.031	1.02 (1.00-1.05)
**Adjustment for additional female-specific factors[c]**					
No. of COPD/person-years	59/26 723	10 188/4 419 880	335/99 983		
Fully-adjusted model[a]	1.08 (0.83-1.39)	ref	1.14 (1.02-1.27)	0.020	1.02 (1.00-1.04)

[a]: Fully-adjusted model: adjusted for age, sex, ethnicity, assessment centres, fasting status, college or university degree, body mass index, smoking status and pack-years, alcohol drinking, summed metabolic equivalent of task-minutes per week for activity, family history of respiratory diseases, prevalent asthma, estimated glomerular filtration rate (eGFR), 25(OH)D, passive smoking, $PM_{2.5}$, occupations associated with COPD.

[b]: The SD was 0.082 mmol/L for albumin-adjusted serum calcium.

[c]: Additional adjustments for female-specific factors included menopause status (no, yes, unknown) and hormone replacement therapy (no, yes, unknown).

Abbreviations: HR, hazard ratio; CI, confidence interval; ref, reference.

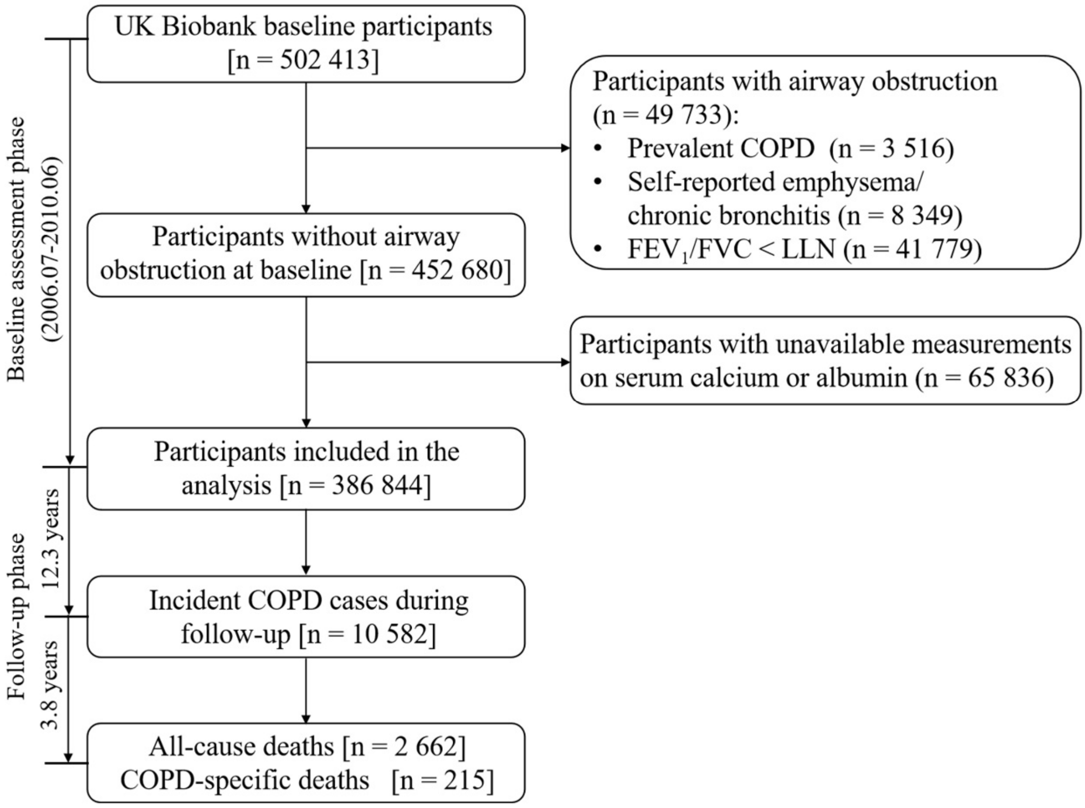

Supplementary Figure S1. Flow chart of study participants.

Abbreviations: COPD = chronic obstructive pulmonary disease; FEV1 = forced expiratory volume in the first second; FVC = forced vital capacity; LLN = lower limit of normal.

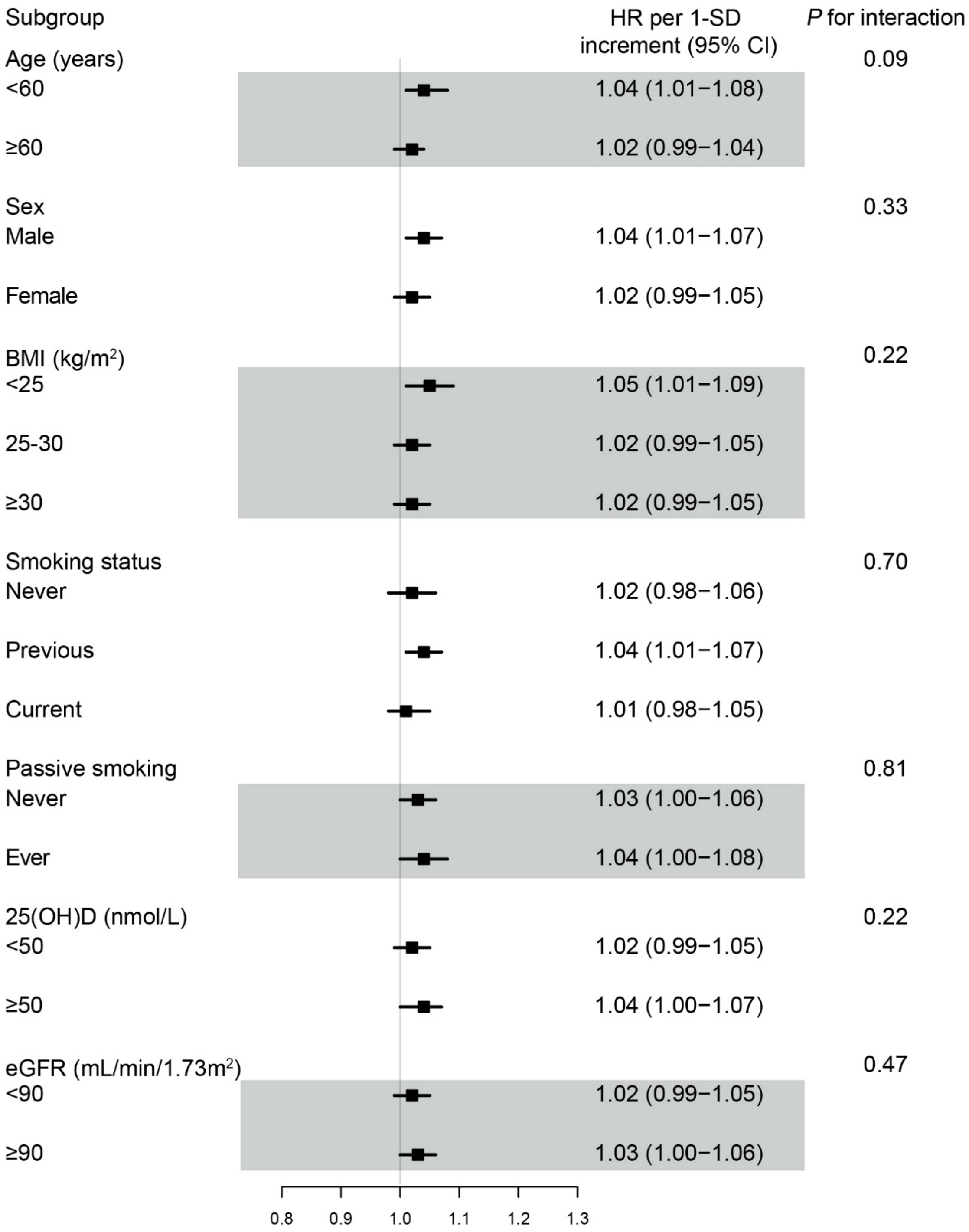

Supplementary Figure S2. Forest plots of stratified analysis of the association between albumin-corrected calcium concentrations and risk of COPD incidence.

Abbreviations: COPD, chronic obstructive pulmonary disease; BMI, body mass index; 25(OH)D, 25-hydroxyvitamin D; eGFR, estimated glomerular filtration rate; HR, hazard ratio; CI, confidence interval.

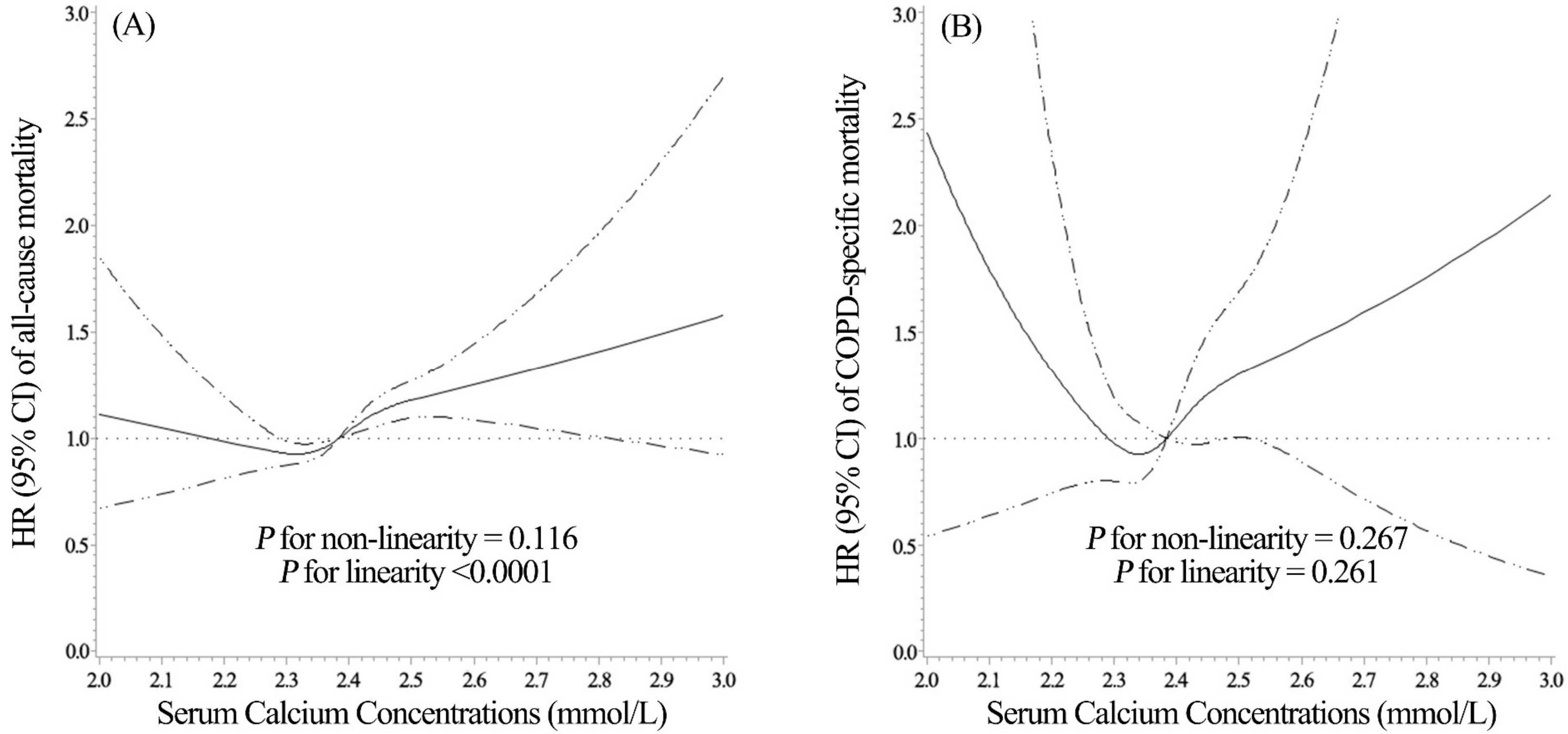

Supplementary Figure S3. Dose-response relationships between albumin-corrected calcium concentrations and the risk of all-cause (A) and COPD-specific (B) mortality in COPD cases. The associations were examined by multivariate cox regression models based on restricted cubic splines. Median serum calcium concentration as a reference. The solid line represents estimates of hazard ratios and the dashed lines represent 95% confidence intervals.
